# Antiwrinkle and antimelanogenesis activity of the ethanol extracts of *Lespedeza cuneata* G. Don for development of the cosmeceutical ingredients

**DOI:** 10.1002/fsn3.682

**Published:** 2018-05-23

**Authors:** Jongsung Lee, Jun Ji, See‐Hyoung Park

**Affiliations:** ^1^ Department of Genetic Engineering Sungkyunkwan University Suwon Korea; ^2^ Department of Natural Medicine Hallym University Chuncheon Korea; ^3^ FA Company Sejong Korea; ^4^ Department of Bio and Chemical Engineering Hongik University Sejong Korea

**Keywords:** antimelanogenesis, antiwrinkle, claudin‐1, *Lespedeza cuneata* G. Don, microphthalmia‐associated transcription factor

## Abstract

To develop the ingredient with the cosmeceutical function, the antiwrinkle and antimelanogenesis effects of the ethanol extract of *Lespedeza cuneata* G. Don were investigated. DPPH radical scavenging activity was significantly increased with the extract of *L. cuneata* G. Don. Cell viability on CCD986Sk human fibroblast was also increased by the ethanol extract of *L. cuneata* G. Don. The inhibitory function of the extract of *L. cuneata* G. Don on collagenase, elastase, and tyrosinase was evaluated. Protein expression level of Claudin‐1, Occludin, and ZO‐1 was up‐regulated in HaCaT human keratinocyte by the extract of *L. cuneata* G. Don. In addition, the extract of *L. cuneata* G. Don inhibited melanin synthesis in B16F10 murine melanoma cells by decreasing MITF, TRP1, and TRP2 protein levels and increasing the phosphorylated Erk and Akt. Thus, these findings would be useful for developing the new cosmeceutical formulations based on the extract of *L. cuneata* G. Don.

## 1 INTRODUCTION

*Lespedeza cuneata* G. Don belongs to Lespedeza and is widely distributed in Japan, China, Taiwan, and Korea (Han, Chung, Nemoto, & Choi, 2010). The biological activity of *L. cuneata* G. Don has been elucidated by several reports demonstrating antiendothelial dysfunction induced by methylglyoxal glucotoxicity (Do et al., 2017), antiaging activity (Seong et al., 2017), anti‐inflammation activity (Lee, Hossaine, & Park, 2016; Lee, Park, et al., 2016), hepatoprotective effect (Kim et al., 2011), and antinitric oxide production activity (Yoo et al., 2015). As a physiologically active substance of *L. cuneata* G. Don, β‐sitosterol, quercetin, kaempferol, pinitol, avicularin, juglanin, and trifolin (Matsuura, Iinuma, Ito, Takami, & Kagei, 1978) have been identified. In addition, the hot water and ethanol extract of *L. cuneata* G. Don have been reported to have antioxidative effect through its flavone glycosides (Deng, Chang, & Zhang, 2007; Park, Kim, & Kwon, 2010). Even though there are a rich of the natural biological resource in *L. cuneata* G. Don, the specific study on the potential of the cosmeceutical composition using *L. cuneata* G. Don is still insufficient. Therefore, the purpose of this study is to investigate whether the ethanol extract of *L. cuneata* G. Don has antiwrinkle and antimelanogenesis activities that are the most interesting aspect of the cosmeceuticals and show the mechanism how it exerts these activities by examining protein‐level working mechanism in skin cells.

Collagenase is a peptidase located in cell membrane and responsible for cleavage of collagen that is a kind of the structural protein for extracellular matrix component (Shoulders & Raines, 2009). Along with collagen, elastin is also another important protein component for the extracellular matrix and elastase is known to break down the elastin protein. Low protein expression level of collagen and elastin is the main cause for the wrinkle formation in skin (Xu, Ryoo, Kim, Choo, & Yoo, 2009). Thus, researcher has tried to develop the novel way to inhibit collagenase and elastase enzyme activity, which might help to provide skin tensile firmness and prepare the cosmeceutical formulation for antiwrinkle and antiaging properties (Apraj & Pandita, 2016).

Tight junction (TJ) is one type of cell junctions and usually can be mediated by the membrane‐integrated proteins such as claudins, occludin, and junctional adhesion molecule (JAM) and the cytoplasmic zona occluden (ZO) proteins (Brandner & Schulzke, 2015; Brandner et al., 2015). Among these proteins, claudins have several types of isomers that are mainly responsible for TJ depending on the tissue types (Shigetomi & Ikenouchi, 2017). Especially, in skin keratinocytes, claudin‐1 is known to be involved in regulation of TJ and its functional mechanism has been studied by several recent reports (Tokumasu, Tamura, & Tsukita, 2017; Tokumasu et al., 2016; Volksdorf et al., 2017). As TJ plays a significant role in skin barrier function, researcher have been considering the novel way for preventing and strengthening skin barrier by the development of various cosmetic materials to control TJ in skin.

Tyrosinase is an oxidase responsible for the conversion of 3,4‐dihydroxyphenylalanine (L‐DOPA) into melanin (D’mello, Finlay, Baguley, & Askarian‐Amiri, 2016) and is regulated by transcriptionally microphthalmia‐associated transcription factor (MITF) (Yamaguchi & Hearing, 2009). MITF can be phosphorylated by Akt or Erk, which decreases MITF protein half‐life through induction of proteasome/ubiquitin‐mediated protein degradation (Im et al., 2017). Melanin synthesis in melanocytes is mediated by the enzymatic reaction of tyrosinase, tyrosinase‐related protein 1 (TRP1), and tyrosinase‐related protein 2 (TRP2) (Shim, Song, Choi, Choi, & Hwang, 2017). The excessive formation of melanin (melanogenesis) in skin can result in hyperpigmentation and even lead to skin cancer (Joo et al., 2015; Lee, Kim, Kim, Heo, & Kim, 1997; Ortonne & Passeron, 2005; Parvez, Kang, Chung, & Bae, 2007). Thus, the novel cosmeceutical ingredients for regulation of melanogenesis will assist skin to be maintained with more healthy status.

In this study, we evaluate the possibility of the ethanol extracts of *L. cuneata* G. Don as a cosmeceutical material. The ethanol extracts of *L. cuneata* G. Don shows the antiwrinkle activity through inhibition of collagenase and elastase as well as induction of up‐regulation of protein markers responsible for TJ in skin. In addition, the ethanol extracts of *L. cuneata* G. Don exerts the antimelanogenesis activity via inhibition of tyrosinase and regulation of the proteins related to controlling tyrosinase expression and activity. In conclusion, our current study might provide evidence that the ethanol extracts of *L. cuneata* G. Don can be applied to a potential agent for treatment of skin disorders by keeping skin tissue maintenance and regulating melanogenesis.

## 2 MATERIALS AND METHODS

### 2.1 Reagents

Dimethyl sulfoxide (DMSO), glycerol, glycine, sodium chloride, Trizma base, vitexin, and Tween20 were from Sigma (St. Louis, MO, USA).

### 2.2 Preparation of the ethanol extract of *Lespedeza cuneata* G. Don

*Lespedeza cuneata* G. Don was collected from the different geographical regions of Korea was rinsed carefully with the fresh water and dried in air. Then, dried *L. cuneata* G. Don was ground and sifted through a 30‐mesh sieve (600 μm, particle size). The powder was extracted with 70% ethanol and the solvent was removed by rotary evaporation.

### 2.3 Cell lines

HaCaT human keratinocyte cell line (ATCC, Manassas, VA, USA), CCD986Sk human fibroblast, and B16F10 murine melanoma cell lines (Korean Cell Line Bank, Seoul, Korea) were maintained in Dulbecco’s modified Eagle’s medium (DMEM) (Thermo Fisher, Waltham, MA, USA) supplemented with 10% fetal bovine serum and 1% streptomycin/penicillin at 37°C in a humidified incubator containing 5% CO2.

### 2.4 Cell viability assay

Cell viability assay was performed with the water‐soluble tetrazolium salts (WST‐1) method. Aliquots of 200 μl of CCD986Sk cell culture (1 × 10^3^ cells/well) were added to a 96‐well plate and incubated for 18 hr. Then, *L. cuneata* G. Don extract at various doses (500, 250, 125, and 62.5 μg/ml) or vitexin (20 μM) were added into each well and cells were incubated for 48 hr. Control cultures were added with DMSO. After incubation, 20 μl of WST‐1 solution (Daeillab Service, Seoul, Korea) was added to each well and cells were incubated for 4 hr. The absorbance of the solution was measured at 460 nm using a microplate reader (Bio‐Rad, Hercules, CA, USA).

### 2.5 Free radical scavenging activity assay

The free radical scavenging activity of the extracts was measured by 2,2‐diphenyl‐1‐picrylhydrazyl (DPPH) method. 20 μl of *L. cuneata* G. Don extract (500, 250, 125, and 62.5 μg/ml) was mixed with 180 μl of DPPH solution (Sigma) in methanol (50 μg/ml) in a 96‐well plate. The plate was stored in the dark for 15 min and the absorbance was measured at 517 nm using the microplate reader.

### 2.6 Collagenase inhibitory assay

Collagenase inhibitory assay was performed using an MMP‐1 Human ELISA kit (Amersham, Little Chalfont, UK) with standard manufacturer’s protocol. 4‐phenylazobezyloxylcarbonyl Pro‐Leu‐Gly‐Pro‐Arg (the synthetic substrate) was dissolved in reaction buffer at 0.3 mg/ml and 250 μl of substrate was added in the reaction tube with 100 μl of various concentration of *L. cuneata* G. Don extract (500, 250, and 125 μg/ml) or vitexin (20 μM). Collagenase was dissolved in buffer at 0.2 mg/ml and 150 μl of enzyme was added in the reaction tube. After incubation at 25°C for 20 min, the reaction was stopped by addition of 6% citric acid. The reaction mixture was separated using 1.5 ml of ethyl acetate. The absorbance was measured at 320 nm using the microplate reader.

### 2.7 Collagen type I synthesis assay

CCD986Sk human fibroblast cells (1 × 10^5^ cells/well) were seeded to a 6‐well plate and cultured for 24 hr. After incubation, the culture medium was changed to serum‐free medium or *L. cuneata* G. Don extract‐treated medium (500, 250, and 125 μg/ml) and incubated for 48 hr. The supernatant was collected from each well and the amount of procollagen type I C‐peptide was measured with a procollagen type I C‐peptide assay kit (Takara, Otsu, Japan) according to manufacturer’s manual.

### 2.8 Elastase activity assay

Elastase activity assay on *L. cuneata* G. Don extract was conducted using Elastase Human ELISA kit (Abcam, Cambridge, UK). To measure elastase activity, 100 μl of 100 mM Tris buffer (pH 8.0), 25 μl of elastase substrate solution, 50 μl of various concentrations of *L. cuneata* G. Don extract (500, 250, and 125 μg/ml) or vitexin (20 μM), and 25 μl of elastase solution were mixed into each well of a 96‐well plate and incubated for 30 min at room temperature. The elastase activity was quantified by measuring light absorbance at 410 nm with the microplate reader.

### 2.9 Tyrosinase activity assay

B16F10 cells were treated with *L. cuneata* G. Don extract (500, 250, and 125 μg/ml) or vitexin (20 μM) for 24 hr and were lysed with lysis buffer (Cell Signaling Technology, Danvers, MA, USA) and then centrifuged at 18,000 g for 30 min at 4°C. The supernatant was collected and tested for the cell tyrosinase activity. The reaction mixture containing 60 mM phosphoric acid buffer solution (pH 6.8), 10 mM L‐DOPA solution, and the supernatant was incubated at 37°C. After incubation, the dopachrome was monitored at 475 nm for 5 min.

### 2.10 Wound healing assay

HaCaT cells (1 × 10^5^ cells/well) were seeded in 6‐well plates and cultured with serum‐free medium for 18 hr. Then, an artificial wound was scratched into the confluent cell using a P200 pipette tip. Microscopy images (Leica DM IL LED; Leica, GmbH, Germany) were taken immediately for record of 0 hr status and then cell culture media was changed with DMEM supplemented with 1% FBS. Cells were treated with the vehicle control or *L. cuneata* G. Don extract (250 μg/ml). Migration of cells was observed and captured by microscopy at the indicated time point (0, 24, 48, 72, and 96 hr).

### 2.11 Melanin content analysis

B16F10 cells (1 × 10^5^ cells/well) were seeded into a 6‐well plate and then incubated for 18 hr. The culture medium was removed and replaced with fresh medium containing various concentrations of *L. cuneata* G. Don extract (500, 250, and 125 μg/ml) or vitexin (20 μM) for 48 hr. After cells were harvested and washed with PBS, total melanin content in cell pellet was imaged with a light microscopy (Nikon1 J1; Nikon, Tokyo, Japan).

### 2.12 Immunoblotting analysis

Cells (CCD986Sk, HaCaT, or B16F10) were treated with various concentrations of *L. cuneata* G. Don extract. Cells were lysed on ice in lysis buffer for 30 min. Total proteins were separated by electrophoresis on 8%–10% SDS polyacrylamide gel and transferred to a PVDF membrane (Merck Millipore, Burlington, MA, USA). Target proteins were detected using the following primary antibodies: anti‐ZO‐1 (1:1000), anti‐Occludin1 (1:1000), anti‐Claudin‐1 (1:1000), anti‐TRP1 (1:1000), anti‐TRP2 (1:1000), anti‐MITF (1:1000), anti‐pErk (1:1000), anti‐Erk (1:1000), anti‐pAkt (1:1000), anti‐Akt (1:1000), and anti‐β‐actin (1:5000), All antibodies were from Cell Signaling Technology. Then, membranes were incubated using and HRP‐conjugated secondary antibody (Jackson Laboratory, Bar Harbor, ME, USA). Chemiluminescence was detected using ECL (Gendepot, Barker, TX, USA).

### 2.13 HPLC analysis of the ethanol extract of *Lespedeza cuneata* G. Don and vitexin

For comparing the retention time of vitexin standard with the crude 70% ethanol extract of *L. cuneata* G. Don, analytical high‐performance liquid chromatography (HPLC) was adopted. Using WATERS SYMMETRY C18 column (WATERS, Seoul, Korea, 4.6 × 220 nm), vitexin standard with the crude 70% ethanol extract of *L. cuneata* G. Don were eluted and analyzed for their retention time. The flow rate of the solvent was 1 ml/min and a single absorption peak was identified at 250 nm. Then, for identification of the active chemical candidates, 15Tesla Fourier transform ion cyclotron resonance (15T FT‐ICR) mass spectrometry analysis was performed.

### 2.14 Statistical analysis

All experiments were performed in triplicate. The data are expressed as the mean ± standard deviation (SD). Significant differences between controls and *L. cuneata* G. Don extract‐treated cells were determined using a Student’s t test at *p* value <0.05.

## 3 RESULTS AND DISCUSSION

### 3.1 Effect of the ethanol extract of *Lespedeza cuneata* G. Don on the human fibroblast viability and DPPH radical scavenging activity

**Figure 1:**
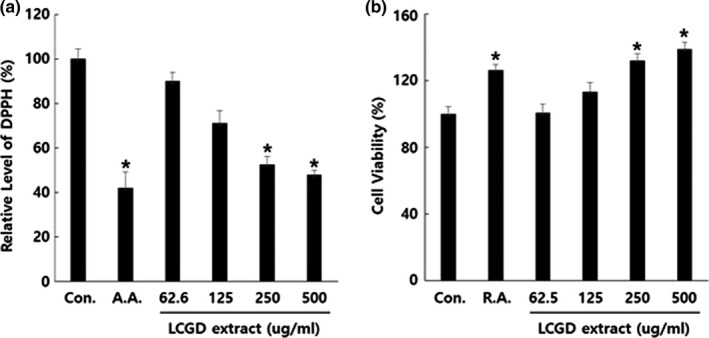
CCD986Sk cell growth activity and antioxidant activity of the ethanol extract of *Lespedeza cuneata* G. Don (a) DPPH radical scavenging capacities of the ethanol extract of *L. cuneata* G. Don compared to the DMSO control. The concentration of DPPH radical was determined spectrophotometrically at 517 nm by a reaction of the ethanol extract of *L. cuneata* G. Don (500, 250, 125, and 62.6 μg/ml) with a 200 μM solution of DPPH. A.A stands for ascorbic acid (1 μM). (b) Effects of the ethanol extract of *L. cuneata* G. Don on the viability of CCD986Sk cells. CCD986Sk cells were treated with 500, 250, 125, and 62.6 μg/ml of the ethanol extract of *L. cuneata* G. Don for 48 hr. Cell viability was assessed with WST‐1 assay. R.A. stands for retinoic acid (1 μM). Error bars indicate the standard deviation (SD). The experimental significance was determined by Student’s t test (*p < 0.05). Experiments were performed in triplicate

Antioxidant effect of the natural plant extracts is well‐known standard to determine whether the extract can be used as a component of the cosmeceutical formulation or not. Antioxidant effect is primarily characterized by the relative amount of flavonoids in each plant (Park, Yang, Kim, & Kim, 2015). As shown in Figure 1a, it was demonstrated that the ethanol extract of *L. cuneata* G. Don showed DPPH radical scavenging activity with dose‐dependent manner. Especially, 250 and 500 μg/ml of the ethanol extract of *L. cuneata* G. Don has the statistically significant (*p* value <0.05) DPPH radical scavenging activity. 500 μg/ml of the ethanol extract of *L. cuneata* G. Don has the similar DPPH radical scavenging activity with 1 μM of ascorbic acid that was used for the positive control.

Then, we investigated the cell growth effect of the ethanol extract of *L. cuneata* G. Don on CCD986Sk human fibroblast cells that are generally known to help skin to be maintained with the proper collagen expression level (Bhagavathula et al., 2009; Ravanti, Heino, López‐OtíN, & Kähäri, 1999). We treated CCD986Sk cells with the ethanol extract of *L. cuneata* G. Don and measured the growth rate of CCD986Sk using the WST‐1 assay. As shown in Figure 1b, the ethanol extract of *L. cuneata* G. Don increased cell growth rate of CCD986Sk with a dose‐dependent manner. 250 and 500 μg/ml of the ethanol extract of *L. cuneata* G. Don has the statistically significant (*p* value <0.05) cell growth effect and the similar activity with 1 μM of retinoic acid that was used for the positive control. Thus, these results might suggest that the ethanol extract of *L. cuneata* G. Don has the potential to provide more collagen around skin fibroblasts without any toxicity.

### 3.2 Measurement of antiwrinkle activity of the ethanol extract of *L. cuneata* G. Don

**Figure 2:**
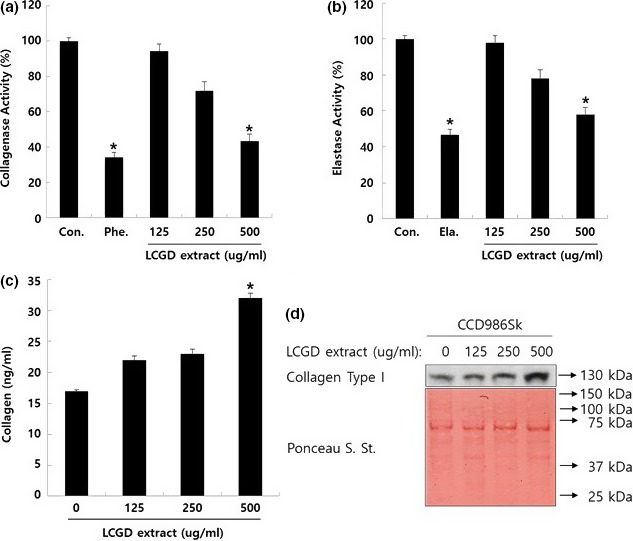
The anticollagenase and elastase activity of the ethanol extract of *Lespedeza cuneata* G. Don (a) Effect of the ethanol extract of *L. cuneata* G. Don on collagenase activity. Collagenase was reacted with various concentrations (500, 250, and 125 μg/ml) of the ethanol extract of *L. cuneata* G. Don as described in materials and methods. 1,10‐phenanthroline (10 μM, abbreviated as Phe.) used as a positive control. (b) Effect of the ethanol extract of *L. cuneata* G. Don on elastase activity. Elastase was reacted with various concentrations (500, 250, and 125 μg/ml) of the ethanol extract of *L. cuneata* G as described in materials and methods. Elastatinal (10 μM, abbreviated as Ela.) was used as a positive control. Error bars indicate the standard deviation (SD). The significance was determined by Student’s t test (*p < 0.05). Experiments were performed in triplicate. (c) Effect of the ethanol extract of *L. cuneata* G. Don on collagen synthesis. CCD986Sk cells were treated with 500, 250, and 125 μg/ml of the ethanol extract of *L. cuneata* G. Don for 24 hr. The collagen contents in culture media were determined by ELISA (d) Western blotting analysis collagen in the culture media. Under the same condition with ELISA analysis, the cell culture media collected and concentrated by Centricon and analyzed by the anticollagen Type I antibody. Ponceau S. staining of the membrane was used for the loading control

The expression of matrix metalloproteinases (MMPs, also called collagenases) are significantly up‐regulated by the external conditions such as UV irradiation and aging (Watanabe et al., 2004). For example, the hyper‐expose of UV irradiation stress to skin fibroblast can promote Erk kinase leading to activation of c‐Jun transcriptional factor. As a result, the expression level of collagenase is increased, which can degrade collagen to induce wrinkle formation around skin dermis (Bhagavathula et al., 2009). Thus, to develop the antiwrinkle ingredients for cosmeceuticals, we investigated the effects of the ethanol extract of *L. cuneata* G. Don on collagenase activity. As shown in Figure 2a, the increased dose of the ethanol extract of *L. cuneata* G. Don resulted in decreased collagenase activity. The collagenase activity was decreased up to approximately 40% compared to the vehicle control at 500 μg/ml of the ethanol extract of *L. cuneata* G. Don, which was the similar value with 10 μM of 1,10‐Phenanthroline that is used for a positive control.

Elastin is another component composing of the extracellular matrix in the connective tissue around skin dermis and can be degraded by elastase, a kind of proteinase enzyme (Meyer, Neurand, & Radke, 1981). Furthermore, hyper elastase activity is known to cause severe immune system‐related diseases such as rheumatoid arthritis and lung fibrosis through the destruction of structural proteins (Dell’aica et al., 2007; Sartor et al., 2002). Therefore, regulation of elastase activity might protect skin connective tissue from losing tissue elasticity and be used for treating way of several diseases. For this purpose, we tried to examine whether the ethanol extract of *L. cuneata* G. Don inhibit elastase activity or not. As shown in Figure 2b, 500 μg/ml of the ethanol extract of *L. cuneata* G. Don decreased elastase activity with an approximate 45% compared to the control. 10 μM of elastatinal was used as a positive control.

Collagen is a kind of extracellular matrix proteins in connective tissues and accounts for about 30% of total proteins in mammals (Di Lullo, Sweeney, Körkkö, Ala‐Kokko, & San Antonio, 2002; Gelse, Pöschl, & Aigner, 2003). There are various kind of collagens in human tissue and Type I collagen is known to play a key role in skin recovery (Seo et al., 2017). Collagen is derived from precursor procollagen that includes the extra‐peptide sequences in N and C‐terminus (Seo et al., 2017). After cleavage of this additional peptide sequence from procollagen by procollagen peptidase, collagens are synthesized as a kind of components for extracellular matrix proteins and incorporated into mesh network of extracellular fibrils (Lee, Hossaine, et al., 2016; Lee, Park, et al., 2016). To examine the amount of collagen in CCD986Sk cell culture media treated by the ethanol extract of *L. cuneata* G. Don, the level of Type I collagen was measured by procollagen Type I C‐peptide ELISA assay kit as the amount of procollagen secreted from CCD986Sk cell reflects the amount of collagen. As shown in Figure 2c, the ethanol extract of *L. cuneata* G. Don increased the expression of Type I collagen with a dose‐dependent way, which was confirmed with Western blotting analysis of Type I collagen collected from the cell culture media under the same condition with ELISA analysis (Figure 2d).

**Figure 3:**
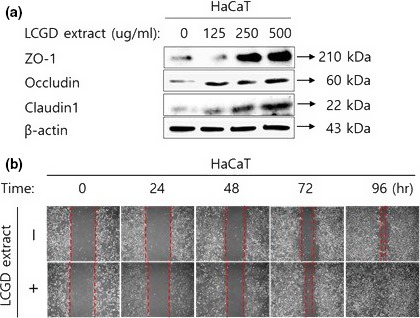
Effect of the ethanol extract of *Lespedeza cuneata* G. Don on protein expression related to TJ in HaCaT cells. (a) Effect of the ethanol extract of *L. cuneata* G. Don on protein expression related to TJ in HaCaT. HaCaT cells were treated with the indicated concentration of the ethanol extract of *L. cuneata* G. Don (500, 250, and 125 μg/ml) for 48 hr. After harvesting cells, ZO‐1, Occludin, and Claudin1 protein expression was examined by Western blotting. β‐actin was used as a loading control. (b) Effect of the ethanol extract of *L. cuneata* G. Don on cell migration of HaCaT. Cells were treated with the ethanol extract of *L. cuneata* G. Don (250 μg/ml). After incubating cells for the indicated time (0, 24, 48, 72, and 96 hr), migration of cells was observed and captured by microscopy at each time point

Adhesive closure functions of TJ are required to maintain skin to be bridged between neighboring cells with the intercellular linkage and to control the movement/permeation of substances between cells (Brandner, 2007). One of the actual effects of the enhanced TJ function in skin is the increase in hydration around skin which would be associated with regulation of wrinkle formation in skin. TJ is mediated with constitutive proteins integrated in the cell membranes and the cytosolic proteins including Claudins, Occludins, JAMs, and ZO‐1. Especially, for TJ in skin, among lots of Claudin isomers, Claudin1 is known to be the most important protein to regulate TJ. Thus, we analyzed the expression level of Claudin1, Occludin, and ZO‐1 in HaCaT human keratinocytes treated with the ethanol extract of *L. cuneata* G. Don by Western blotting. As shown in Figure 3a, Claudin1, Occludin, and ZO‐1 was up‐regulated by the ethanol extract of *L. cuneata* G. Don in HaCaT cells, which was confirmed by wound healing migration assay as the enhanced TJ function can affect cell mobility and migration (Volksdorf et al., 2017) (Figure 3b). The treatment of 250 and 500 μg/ml of the ethanol extract of *L. cuneata* G. Don has significantly increased the expression of Claudin1, Occludin, and ZO‐1 compared to the vehicle control. After 24 hr incubation, 250 μg/ml of the ethanol extract of *L. cuneata* G. Don induced HaCaT cells to migrate more faster into the center of the wound. Therefore, these results might imply that the ethanol extract of *L. cuneata* G. Don can be used for one of the skin hydration cosmeceutical ingredients through up‐regulation of TJ in skin.

### 3.3 Measurement of antimelanogenesis activity of the ethanol extract of *Lespedeza cuneata* G. Don

**Figure 4:**
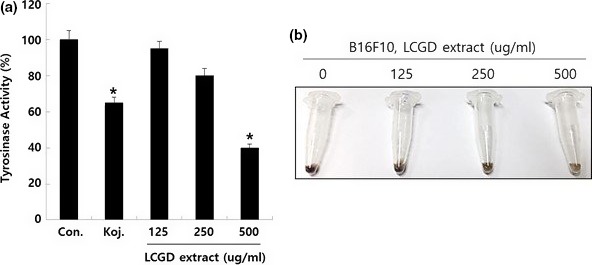
Effect of the ethanol extract of *Lespedeza cuneata* G. Don on melanin synthesis. (a) The antityrosinase activity of the ethanol extract of *L. cuneata* G. Don Tyrosinase was reacted with various concentrations (500, 250, and 125 μg/ml) of the ethanol extract of *L. cuneata* G. Don as described in materials and methods. Kojic acid (10 μM, abbreviated as Koj.) was used as a positive control. Error bars indicate the standard deviation (SD). The significance was determined by Student’s t test (*p < 0.05). Experiments were performed in triplicate. (b) Evaluation of melanin content in B16F10 after treatment of the ethanol extract of *L. cuneata* G. Don with the indicated concentrations (500, 250, and 125 μg/ml) for 48 hr. After cells were harvested and washed with PBS, total melanin content in cell pellet was imaged with a light microscopy

As the initial enzyme for melanin synthesis, tyrosinase plays a key role in pigmentation of skin and hair as well as in wound healing and immune response (Cabanes, Chazarra, & Garcia‐Carmona, 1994; Fairhead & Thöny‐Meyer, 2012). Tyrosinase is known to be induced by ionizing and UV radiation exposure to activate melanogenesis that takes place in melanosomes of melanocyte (Tsatmali, Ancans, & Thody, 2002). Excessive melanogenesis is generally associated with the pigmentation‐related disorders such as hyperpigmentation and skin cancer (Costin & Hearing, 2007; Yoon et al., 2015). Tyrosinase inhibitors could be used for the preventing skin hyperpigmentation and the treatment of skin disorders (Ortonne & Passeron, 2005; Parvez et al., 2007). We tried to elucidate the effect of the ethanol extract of *L. cuneata* G. Don on tyrosinase activity. As shown in Figure 4a, 500 μg/ml of the ethanol extract of *L. cuneata* G. Don inhibited tyrosinase activity with about 60% compared to the control. 10 μM of Kojic acid was used for the positive control and showed approximately 40% inhibition of tyrosinase activity. In addition, to confirm the antimelanogenesis activity of the ethanol extract of *L. cuneata* G. Don, we examined pigment formation in B16F10 murine melanoma cells treated by the ethanol extract of *L. cuneata* G. Don. As shown in Figure 4b, the pigmentation of B16F10 cell pellets was significantly decreased by the ethanol extract of *L. cuneata* G. Don. Therefore, their results imply that the ethanol extract of *L. cuneata* G. Don has the potential to be developed as cosmeceutical formulations to prevent the overproduction of melanin in skin.

**Figure 5:**
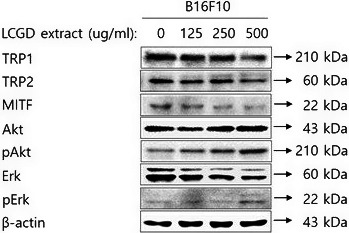
Effect of the ethanol extract of *Lespedeza cuneata* G. Don on protein expression related to melanin synthesis in B16F10 cells. B16F10 cells were treated with the indicated concentration of the ethanol extract of *L. cuneata* G. Don (500, 250, and 125 μg/ml) for 48 hr. After harvesting cells, TRP1, TRP2, MITF, pAkt, Akt, pErk, and Erk protein expression was examined by Western blotting. β‐actin was used as a loading control

TRP1 and TRP2 have about 40% homology with tyrosinase, are also responsible for melanogenesis of melanocytes, and transcriptionally modulated by MITF, a major transcription factor in melanogenesis (Slominski, Tobin, Shibahara, & Wortsman, 2004; Yasumoto, Yokoyama, Takahashi, Tomita, & Shibahara, 1997; Yavuzer et al., 1995). TRP1 is involved in regulation of tyrosinase activity by stabilizing of tyrosinase protein (Ghanem & Fabrice, 2011) and in affecting melanocyte proliferation by maintenance of melanosome structure (Sarangarajan & Boissy, 2001). TRP2 (dopachrome tautomerase) mediates cyclization of L‐DOPA resulting in synthesis of dopachrome, one of the intermediates during the biosynthesis of melanin (Slominski et al., 2004). To investigate whether the ethanol extract of *L. cuneata* G. Don can affect the expression level of melanogenic proteins such as TRP1, TRP2, and MITF, we treated B16F10 cells with the ethanol extract of *L. cuneata* G. Don and performed Western blotting analysis for those proteins. As shown in Figure 5, the expression level of TRP1 and TRP2 were significantly downregulated with 500 μg/ml of the ethanol extract of *L. cuneata* G. Don. The expression level of MITF was decreased in a dose‐dependent manner by the ethanol extract of *L. cuneata* G. Don. Taken together, these results suggest that the ethanol extract of *L. cuneata* G. Don may inhibit melanin synthesis in B16F10 cells by downregulating protein expression of MITF and its target proteins.

We further investigated whether the ethanol extract of *L. cuneata* G. Don on the reduction in MITF is related to the phosphorylation of Akt and Erk. The activation of Akt and Erk signaling pathways has been reported to inhibit melanogenesis (Englaro et al., 1998; Kim et al., 2003; Lee, Jung, Kim, & Park, 2007; Oka et al., 2000). Both of Akt and Erk activation can phosphorylate MITF followed by proteasome‐mediated ubiquitination leading to decrease in MITF protein stability and activation of MITF protein degradation. Thus, to determine the specific mechanism involved in inhibition of melanin synthesis by the ethanol extract of *L.  cuneata* G. Don, we investigated the effect of the ethanol extract of *L. cuneata* G. Don on the phosphorylation of Akt and Erk in B16F10 melanoma cells by Western blotting analysis. As shown in Figure 5, treatment of the ethanol extract of *L. cuneata* G. Don up‐regulated dramatically the expression level of the phosphorylated Akt with dose‐dependent manner and Erk at the 500 μg/ml of the ethanol extract of *L. cuneata* G. Don. These findings indicate that the inhibitory activity of the ethanol extract of *L. cuneata* G. Don against melanogenesis may be associated with the activation of Erk and Akt pathways causing the induction of proteasomal degradation of MITF.

### 3.4 Identification of vitexin from 70% ethanol extract of *Lespedeza cuneata* G. Don and evaluation of vitexin for antiwrinkle and antimelanogenesis activity

**Figure 6:**
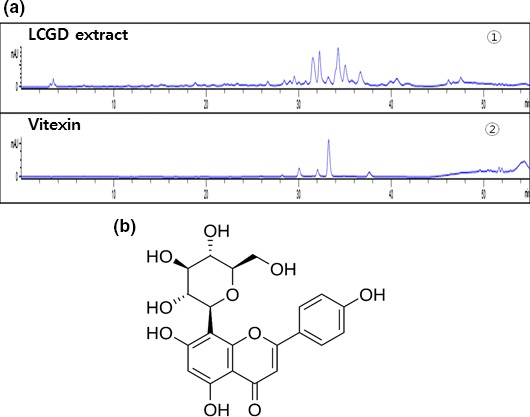
Identification of vitexin from 70% ethanol extract of *Lespedeza cuneata* G. Don. (a) High‐performance liquid chromatography (HPLC) profile of 70% ethanol extract of *L. cuneata* G. Don and the vitexin standard. For comparing the retention time of the vitexin standard with the crude 70% ethanol extract of *L. cuneata* G. Don, analytical HPLC was adopted. (b) Chemical structure of vitexin, a kind of flavonoids

**Figure 7:**
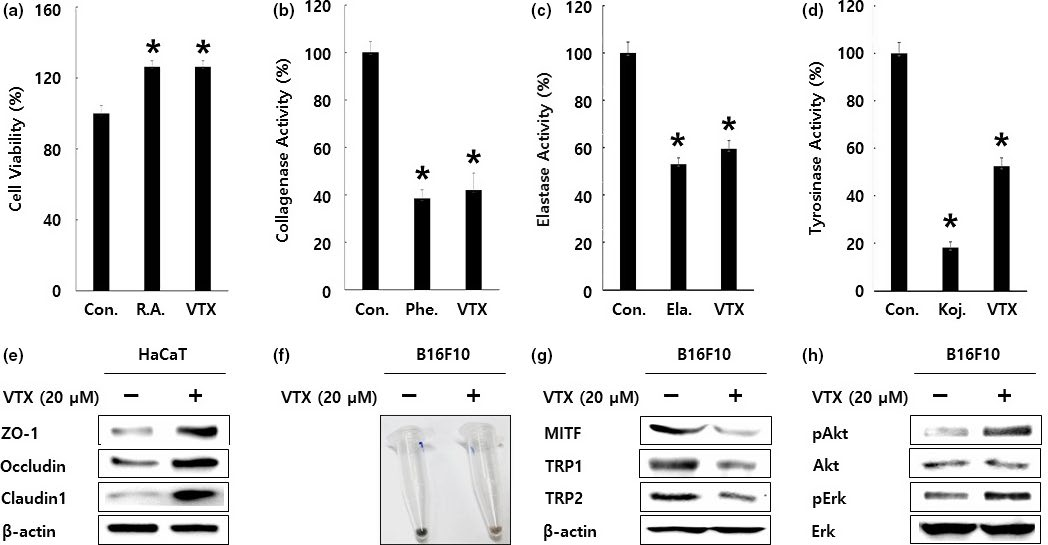
Antiwrinkle and antimelanogenesis activity of vitexin. (a) Stimulation of CCD986Sk human fibroblast growth by vitexin (VTX, 20 μM). Retinoic acid (R.A., 1 μM) was used for the positive control. Inhibitory activity of vitexin (VTX, 20 μM) against collagenase (b), elastase (c), and tyrosinase (d). Phenanthroline (10 μM, abbreviated as Phe.) used as a positive control for collagenase assay. Elastatinal (10 μM, abbreviated as Ela.) was used as a positive control for elastase assay. Kojic acid (10 μM, abbreviated as Koj.) was used as a positive control for tyrosinase assay. Error bars indicate the standard deviation (SD). The experimental significance was determined by Student’s t test (*p < 0.05). Experiments were performed in triplicate. (e) Up‐regulation of TJ‐related protein expression in HaCaT human keratinocytes by vitexin (VTX, 20 μM). β‐actin was used as a loading control. (f) Inhibition of the melanin synthesis in B16F10 murine melanoma cells by vitexin (VTX, 20 μM). (g) Regulation of the melanin synthesis‐related protein expression in B16F10 murine melanoma cells by vitexin (VTX, 20 μM). β‐actin was used as a loading control

To show the bioactive components responsible for antiwrinkle and antimelanogenesis activity, we performed analytical HPLC analysis and 15Tesla Fourier transform ion cyclotron resonance (15T FT‐ICR) mass spectrometry analysis. As shown in Figure 6a,b, vitexin, a kind of flavonoids, was successfully identified from 70% ethanol extract of *L. cuneata* G. Don. Then, we evaluated the antiwrinkle and antimelanogenesis activity of vitexin by in‐vitro enzyme assays and western blotting analysis. As shown in Figure 7, vitexin showed the cell growth effect for CCD986Sk human fibroblast (A) and the inhibitory activity against collagenase (B), elastase (C), and tyrosinase (D) at the 20 μM. Furthermore, vitexin caused the up‐regulation of Claudin‐1, Occludin, and ZO‐1 in HaCaT human keratinocyte (Figure 7e). And vitexin reduced the melanin synthesis in B16F10 murine melanoma cells (Figure 7f), decreased the protein expression of MITF, TRP1, and TRP2, and increased the phosphorylation of Erk and Akt in B16F10 murine melanoma cells (Figure 7g). These results suggested that vitexin from 70% ethanol extract of *L. cuneata* G. Don might be responsible for antiwrinkle and antimelanogenesis activity of 70% ethanol extract of *L. cuneata* G. Don.

## 4 CONCLUSION

Natural components derived from extracts of traditional herbal plant are of interest for cosmetic applications such as antiwrinkle and skin whitening. As the potential of the functional ingredients for cosmeceuticals, the novel activity of the ethanol extract of *L. cuneata* G. Don is not still fully understood and molecular mechanisms related to its activity remains to be discovered. For this purpose, we demonstrate that the ethanol extracts of *L. cuneata* G. Don stimulate CCD986Sk human fibroblast growth with the DPPH radical scavenging activity. And the ethanol extracts of *L. cuneata* G. Don induce TJ function‐related proteins expression as well as cell migration of HaCaT human keratinocyte. Furthermore, we found the inhibitory effects of the ethanol extracts of *L. cuneata* G. Don on wrinkle formation through up‐regulation of collagen synthesis in CCD986Sk human fibroblast cells and melanogenesis in B16F10 melanoma cells through downregulation of MITF, TRP1, and TRP2. Thus, to the best of our knowledge, the current study provides the first evidences that as safe and effective ingredients, the ethanol extract of *L. cuneata* G. Don possess the protective effects against damage in skin connective tissues. Although we focused on the possibility for cosmetic agents using the ethanol extract of *L. cuneata* G. Don, as we mentioned in the introduction part, *L. cuneata* G. Don has been traditionally used as the medicinal herbal food in Asia for treating various diseases and symptom. In this study, we performed in‐vitro assays for showing the novel cosmeceutical activity such as antiwrinkle and antimelanogenesis and suggested *L. cuneata* G. Don, a kind of traditionally edible herbal plants, could be used for agents for cosmeceutical product. Thus, we think that this study will contribute to the expansion of the application using the traditional herbal food to another industrial and scientific field. For the further studies, we plan to identify the more active compounds separated from the ethanol extract of *L. cuneata* G. Don and elucidate the detailed relationship between the candidate compounds and signaling pathways for collagen synthesis, TJ activation, and melanogenesis. In conclusion, our study could help to develop new cosmetic and pharmaceutical substances based on the ethanol extract of *L. cuneata* G. Don.

## CONFLICT OF INTEREST

The author declares no conflict of interest.

## ETHICAL STATEMENT

Neither animal nor human testing was not involved in this study.
